# Changes in central and peripheral hemodynamic parameters during blood donation

**DOI:** 10.3389/fcvm.2025.1628366

**Published:** 2025-09-23

**Authors:** Babak Yazdani, Gökhan Yücel, Katrin Schulz, Sabine Kayser, Behnam Shaygi, Gerhard Schumacher, Janine Poschauko, Bernhard K. Krämer, Daniel Duerschmied, Anna Hohneck

**Affiliations:** ^1^ Fifth Department of Medicine, University Medical Center Mannheim UMM, Faculty of Medicine of the University of Heidelberg, Mannheim, Germany; ^2^ First Department of Medicine, University Medical Center Mannheim UMM, Faculty of Medicine of the University of Heidelberg, Mannheim, Germany; ^3^ European Center for AngioScience (ECAS) and German Center for Cardiovascular Research (DZHK) Partner Site Heidelberg/Mannheim, Mannheim, Germany; ^4^ Medical Clinic V, Nephrology, Rheumatology, Blood Purification, Academic Teaching Hospital Braunschweig, Braunschweig, Germany; ^5^ Institute of Transfusion Medicine and Immunology, Medical Faculty Mannheim, Heidelberg University, German Red Cross Blood Service Baden-Württemberg-Hessen, Mannheim, Germany; ^6^ Department of Interventional Radiology, London North West University Healthcare NHS Trust, London, United Kingdom; ^7^ inmediQ GmbH, Butzbach, Germany; ^8^ First Department of Medicine, KRH Klinikum Robert Koch Gehrden, Gehrden, Germany; ^9^ Department of Endocrinology, Diabetology, Metabolism and Clinical Chemistry, Heidelberg University Hospital, Heidelberg, Germany

**Keywords:** aortic blood pressure, pulse wave analysis, heart rate, vascular resistance, cardiovascular physiological phenomena, augmentation index (AIx), left ventricular ejection time (LVET)

## Abstract

**Background:**

Blood donation is a common procedure, yet its acute effects on central and peripheral hemodynamics are not fully understood. This study aimed to systematically quantify immediate cardiovascular changes induced by whole blood donation in healthy adults, with a primary focus on arterial pressure and central hemodynamic parameters.

**Methods:**

Thirty healthy volunteers (12 female, 18 male; median age 34 years, IQR 24–53) underwent standardized whole blood donation. Non-invasive measurements of central and peripheral hemodynamics were performed immediately before and after donation using the VascAssist 2 device, enabling assessment of brachial and aortic blood pressures, heart rate, augmentation index (AIx), pulse wave velocity (PWV), and left ventricular ejection time (LVET).

**Results:**

Blood donation resulted in a significant reduction in brachial systolic blood pressure (SBP) [pre: 131 mmHg [IQR 121–138] vs. post: 127 mmHg [IQR 116–134]; median change −4mmHg, IQR −10 to 0; *p* = 0.002]. No statistically significant changes were observed in heart rate [pre: 72 bpm [IQR 68–80] vs. post: 74 bpm [IQR 64–81]; *p* = 0.82], diastolic blood pressure (DBP) [pre: 75 mmHg [IQR 68–81] vs. post: 74 mmHg [IQR 70–85]; *p* = 0.66], aortic SBP, or central PWV. Significant reductions were observed in augmentation index [AIx75: pre 1% [IQR −9 to 6] vs. post −5% [IQR −11 to 4]; *p* = 0.02] and LVET [pre: 244 ms [IQR 225–257] vs. post: 231 ms [IQR 215–243]; *p* = 0.0005]. No statistically significant correlations were identified between these hemodynamic responses and sex, age, body mass index, or hemoglobin concentration.

**Conclusion:**

Acute whole blood donation induces a mild but statistically significant decrease in peripheral SBP, accompanied by reductions in AIx and LVET, while central aortic blood pressure and vascular stiffness remain unchanged. These findings indicate that healthy individuals exhibit immediate adaptive mechanisms that preserve central cardiovascular stability in response to mild volume depletion. The results support the overall hemodynamic tolerability of blood donation and provide insight into the transient vascular and cardiac adaptations elicited by acute blood loss.

## Introduction

Blood donation remains a cornerstone of modern medicine, yet acute cardiovascular responses to this procedure are not fully understood at the hemodynamic level (1, 2). While most blood donations proceed without serious complications, vasovagal reactions and transient disturbances in blood pressure and consciousness occur, particularly among younger donors (3). Strategies such as pre-donation hydration and muscle tension have been shown to reduce vasovagal episodes (4). However, the underlying circulatory adaptations, including possible cerebral and arterial compensatory mechanisms, are still being elucidated.

Previous research has demonstrated that acute blood loss can lead to reductions in systolic, diastolic, and mean arterial blood pressure, as well as stroke volume, while cardiac output may be maintained through compensatory increases in heart rate (5). Cerebral vasodilation has also been observed, likely as a means of preserving cerebral perfusion in the setting of mild hypovolemia (6). Nonetheless, most studies have focused on post-donation measurements or evaluated hemodynamic changes under conditions of exertion, limiting insights into the immediate hemodynamic dynamics during donation itself.

Accurate assessment of central hemodynamic changes is challenging, as invasive measurements are not feasible in routine or research settings involving healthy volunteers. Non-invasive pulse wave analysis (PWA), particularly via oscillometric or tonometric devices, enables estimation of central blood pressure and arterial stiffness (e.g., pulse wave velocity (PWV), augmentation index (AIx) from peripheral recordings (7, 8). However, existing technologies such as SphygmoCor are subject to inter-operator variability and calibration errors.

The VascAssist 2 device, which employs radial oscillometric PWA coupled with a validated computational arterial model, enables operator-independent assessment of both peripheral and central hemodynamic parameters, including brachial and aortic blood pressures, PWV, AIx, and left ventricular ejection time (LVET) (9–11). This technology allows for continuous and reproducible hemodynamic assessments even in the context of arrhythmias or limited technical skill (12).

In a cohort of more than 3,000 individuals at intermediate to high cardiovascular risk, neither invasively measured central blood pressure nor non-invasively measured brachial blood pressure independently predicted mortality (13). Recent work has highlighted the relevance of non-invasive central hemodynamic measurements in both clinical and physiological research settings (14, 15). Moreover, current reviews emphasize that augmentation index and arterial stiffness are highly dynamic parameters influenced by preload conditions (16, 17). In the context of acute blood volume shifts, recent physiological studies using near-infrared spectroscopy confirm robust compensatory cerebral and systemic mechanisms, thereby underlining the importance of integrated monitoring approaches (18, 19).

Based on this background, the present study aimed to characterize the immediate changes in central and peripheral hemodynamic parameters during whole-blood donation in healthy adults using non-invasive model-based PWA. Our primary objective was to determine the effect of blood donation on arterial pressure, anticipating a reduction in peripheral (brachial) systolic blood pressure, whereas central hemodynamic parameters were expected to remain relatively preserved as a result of rapid compensatory mechanisms.

Secondary aims included characterization of changes in heart rate, AIx, and arterial stiffness, as well as exploration of the influence of age, sex, body mass index (BMI) and hemoglobin (Hb) levels on individual hemodynamic responses.

## Methods

This prospective single-center study was conducted at the University Medical Center Mannheim, University of Heidelberg, Germany between May and August 2024. The study enrolled 30 healthy adult volunteers (12 women, 18 men; median age: 34 years, IQR 24–53) slated for routine whole blood donation. All participants provided written informed consent. The study was approved by the institutional ethics committee [Medical Ethics Commission II, Faculty of Medicine Mannheim, University of Heidelberg, Germany; 2021−410M-§ 47 (3) MPDG] and conducted in accordance with the Declaration of Helsinki. Data were protected in accordance with the EU Data Protection Directive.

Participants were eligible if they met the following criteria: age 18–68 years (first-time donors under 60), self-reported good health, minimum body weight of 50 kg, and Hb concentration of ≥12.5 g/dl for women or ≥13.5 g/dl for men. Blood pressure at screening was required to be within 100–160 mmHg systolic and 60–100 mmHg diastolic. Resting pulse rate between 50 and 110 bpm was mandatory, elite or competitive athletes, defined as those with regular participation in high-level competitions, were excluded to minimize confounding from pronounced athletic cardiovascular adaptations.

Absolute exclusion criteria encompassed: inability to provide informed consent; age <18 years; HIV or hepatitis C infection; clinically significant cardiovascular disease; severe disorders of the central nervous system; relevant coagulopathies; malignant neoplastic disease; severe chronic disease of the kidneys, lungs, or digestive system; autoimmune disorders such as Hashimoto's thyroiditis; insulin-dependent diabetes mellitus; presence of tremor; bilateral vascular access, axillary dissection, or amputation; regular use of medications affecting cardiovascular function; or pregnancy.

### Hemodynamic assessment

Hemodynamic parameters, including brachial and aortic blood pressures, heart rate, augmentation index (AIx adjusted to a heart rate of 75 bpm, AIx75), PWV, and LVET, were determined non-invasively using the VascAssist 2 device (inmediQ GmbH, Germany). Assessments were performed according to manufacturer recommendations and standardized participant positioning. Measurements were obtained within 10 min before and 10 min after donation of 450 ml whole blood.

### Statistical analysis

Continuous variables are reported as median (interquartile range, IQR). Comparisons between pre- and post-donation values were performed using the Wilcoxon signed-rank test for paired samples. Correlations between changes in hemodynamic parameters and participant characteristics (age, sex, BMI, Hb) were assessed using Spearman's rank correlation coefficient. A two-tailed *p*-value <0.05 was considered statistically significant. Statistical analyses were conducted using GraphPad Prism 10.4.1 (GraphPad Software, Inc., San Diego, California). All analyses are of exploratory nature.

As the primary endpoint was the change in brachial systolic blood pressure, a sample size calculation was performed using G*Power 3.1 (Heinrich Heine University Düsseldorf, Germany). Assuming a medium effect size (Cohen's d = 0.5) based on reported acute post-donation SBP changes (20), a two-sided *α* = 0.05, and a power of 0.8, a minimum of 27 participants was required. We therefore included 30 individuals, which was considered sufficient to detect clinically relevant changes in the primary endpoint.

## Results

**Figure 1:**
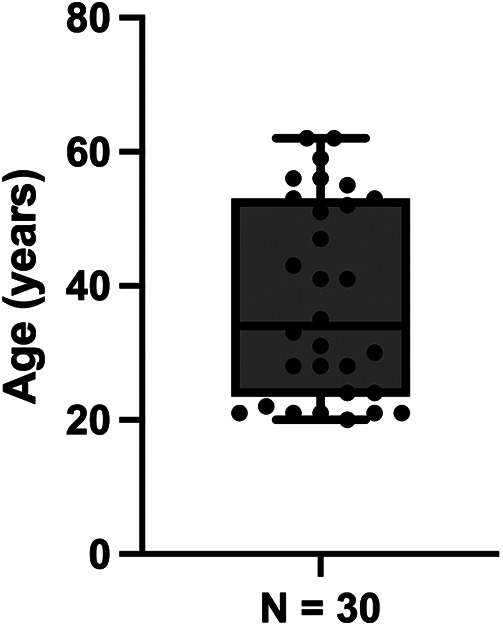
Age distribution of participants. Age distribution of the total patient cohort included in the study (*n* = 30).

Thirty healthy adults (12 female, 18 male) were included in the analysis. The median age was 34 years [interquartile range (IQR): 24–53; minimum: 20; maximum: 62], with the third quartile at 53 years, indicating that while the cohort skewed younger, a substantial proportion of participants were middle-aged (see Figure 1 for detailed age distribution). The median body mass index (BMI) was 25 kg/m^2^ (IQR: 24–30). Baseline brachial systolic blood pressure (SBP) in the study population was 131 mmHg (median; IQR: 121–138 mmHg), and brachial diastolic blood pressure (DBP) was 75 mmHg (median; IQR: 68–81 mmHg). The median resting heart rate was 72 bpm (IQR: 68–80 bpm). Pre-donation Hb concentration had a median value of 14.1 g/dl (IQR: 13.7–15.0 g/dl). All participants fulfilled the predefined eligibility criteria, and no elite competitive athletes were enrolled. Complete baseline characteristics can be obtained from Table 1.

**Table 1:** Baseline characteristics.

Baseline patient characteristics	Number of patients (*N* = 30)
Sex, female (%)	12 (40.0)
Age, years	34 (24; 53)
Height, cm	175 (167; 180)
Weight, kg	79 (71; 89)
BMI, kg/m^2^	25 (24; 30)
Brachial SBP, mmHg	131 (121; 138)
Brachial DBP, mmHg	75 (68; 81)
Heart rate, bpm	72 (68; 80)
Hb, g/dl	14.1 (13.7; 15.0)

Data are presented as median (IQR) or frequencies.

BMI, body-mass-index; SBP, systolic blood pressure; DBP, diastololic blood pressure; bpm, beats per minute; Hb, hemoglobin.

**Figure 2:**
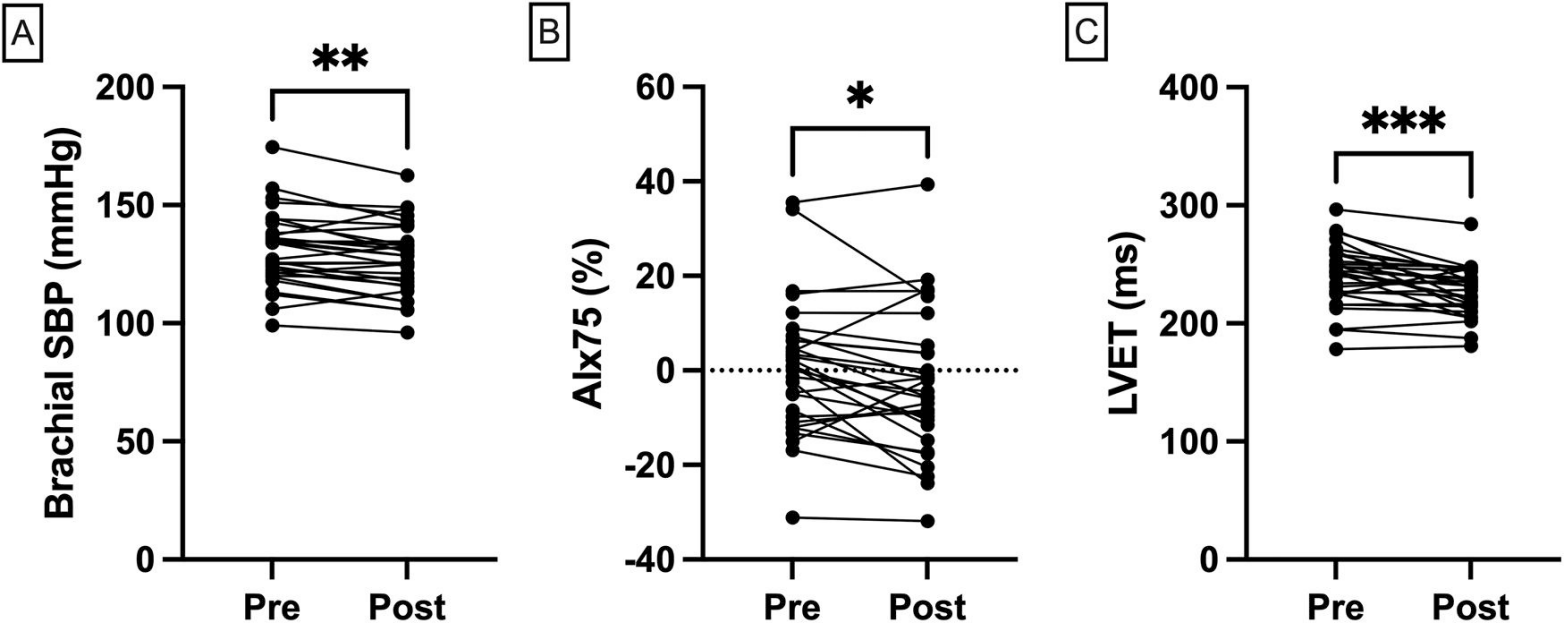
Hemodynamic changes following blood donation. Individual pre- and post-donation trajectories comparing changes in **(A)** brachial SBP; **(B)** AIx75; and **(C)** LVET, assessed by VascAssist 2 device. AIx, augmentation index; LVET, left ventricular ejection time, SBP, systolic blood pressure.

Peripheral and central hemodynamic parameters were assessed before and after blood donation. Brachial SBP decreased significantly from a median of 131 mmHg (IQR 121–138) pre-donation to 127 mmHg (IQR 116–134) post-donation (median change: −4 mmHg, IQR: −10 to 0; *p* = 0.002; Figure 2; Table 2). No statistically significant difference was found in brachial DBP [pre: 75 mmHg [IQR 68–81] vs. post: 74 mmHg [IQR 70–85], *p* = 0.66], or in heart rate [pre: 72 bpm [IQR 68–80] vs. post: 74 bpm [IQR 64–81], *p* = 0.82]. It should be noted that the absence of statistical significance does not exclude the possibility of physiologically relevant changes, particularly as the study was not powered to detect small differences in these parameters. Central hemodynamics, as assessed by aortic SBP [pre: 106 mmHg [IQR 99–114] vs. post: 107 mmHg [IQR 98–113], *p* = 0.09] and central PWV [pre: 7.6 m/s [IQR 6.8–8.8] vs. post: 8.2 m/s [IQR 7.0–9.0], *p* = 0.25], likewise exhibited no statistically significant post-donation changes. AIx75 demonstrated a significant reduction post-donation [pre: 1% [IQR −9 to 6] vs. post: −5% [IQR −11 to 4], *p* = 0.02]. LVET was also significantly lower after donation [pre: 244 ms [IQR 225–257] vs. post: 231 ms [IQR 215–243], *p* = 0.0005], which reflects a shortening of systole duration (Figure 2; Table 2).

**Table 2:** Central and peripheral hemodynamic parameters.

Hemodynamic parameters	Before blood donation	After blood donation	*p*-value
Heart rate, bpm	72 (68; 80)	74 (64; 81)	0.82
Brachial SBP, mmHg	131 (121; 138)	127 (116; 134)	**0** **.** **002**
Brachial DBP, mmHg	75 (68; 81)	74 (70; 85)	0.66
Central PWV, m/s	7.6 (6.8; 8.8)	8.2 (7.0; 9.0)	0.25
Stiffness, %	25 (17; 49)	27 (17; 39)	0.20
Resistance, %	34 (17; 49)	26 (18; 47)	0.18
Aortic SBP, mmHg	106 (99; 114)	107 (98; 113)	0.09
Aortic DBP, mmHg	77 (67; 83)	77 (69; 86)	0.41
AIx75, %	1 (−9; 6)	−5 (−11; 4)	**0**.**02**
LVET, ms	244 (225; 257)	231 (215; 243)	**0**.**0005**

Data are presented as median (IQR). Bold values mark statistical significance.

bpm, beats per minute; SBP, systolic blood pressure DBP, diastolic blood pressure; PWV, pulse wave velocity; AIx75, augmentation index adjusted to a heart rate of 75 beats per minute; LVET, left ventricular ejection time.

Individual pre- and post-donation trajectories for core hemodynamic parameters are depicted in Figures 2A–C.

### Correlation analyses

No statistically significant correlations were found between age, sex, BMI or Hb and the observed hemodynamic changes (Supplementary Table S1). Women had a higher heart rate at rest [*r* = 0.37 (0.02; 0.65), *p* = 0.04], while AIx was inversely correlated with height [−0.44 (−0.69; −0.10), *p* = 0.01], which was physiologically expected (Supplementary Figures S1, S2).

### Adverse events

No adverse events (including symptomatic hypotension, presyncope, or vasovagal episodes) were recorded during or immediately after blood donation.

## Discussion

Hypertension is associated with vascular remodeling and increased arterial stiffness in small, medium and large arteries, mediated by mechanical, inflammatory, and neurohumoral factors, as highlighted by the PAMELA study (21). However, brachial and central blood pressure may differ, while brachial blood pressure significantly overestimates carotid arterial stiffness compared to central blood pressure, particularly in individuals with hypertension (22). Non-invasive estimation of aortic blood pressure shows systematic differences compared with invasive measurements, with carotid and brachial recordings providing the closest agreement. Calibration method, recording site, and waveform analysis critically influence accuracy, highlighting the need for methodological standardization (23).

This study provides a detailed hemodynamic profile of healthy adults during acute whole-blood donation, uniquely combining simultaneous peripheral and central vascular measurements with paired individual pre/post data. Unlike much of the prior research, which has predominantly focused on adverse events or indirect markers of cardiovascular adaptation such as symptoms or basic vital signs, our study utilizes model-based pulse wave analysis to capture subtle changes in brachial and aortic pressures, AIx, PWV, and LVET. Thus, it contributes new quantitative insight into both vascular and cardiac responses during mild, controlled blood loss.

Consistent with earlier reports, a statistically significant decrease in peripheral (brachial) SBP was observed following donation, whereas central aortic blood pressure and central PWV did not change significantly.

These findings align with previous investigations (20), demonstrating that acute reductions in blood volume tend to produce modest decreases in peripheral systolic pressures, often without measurable effects on central hemodynamics. Unlike these earlier studies, our work directly compares these phenomena in the same individuals and over immediate pre/post periods using a non-invasive, operator-independent device.

A key aspect of our hypothesis was that central hemodynamic stability would be preserved via rapid compensatory mechanisms. One principal mechanism involves a shift of interstitial fluid into the vascular compartment, a process that can partially restore plasma volume within minutes after acute blood loss. Classic studies have shown that this process occurs as a consequence of transient reductions in capillary hydrostatic pressure, leading to increased reabsorption of interstitial fluid (Starling forces). Within 20–60 min after blood loss, up to 80% of the lost plasma volume may be replaced by such fluid shifts, provided the individual is euvolemic and healthy (24–26). This mechanism, alongside baroreflex-mediated autonomic adjustments, underpins the maintenance of central pressure despite a measurable fall in peripheral pressure. Although central systolic blood pressure did not change significantly (*p* = 0.09), the result was close to the predefined significance threshold. Given the small sample size, the possibility of a type II error cannot be excluded. More frequent intra-donation measurements might have detected transient central reductions, which could have been masked by rapid interstitial fluid shifts or baroreflex activation. Thus, central pressure responses to acute blood loss may be more dynamic than captured within our single pre/post measurement design.

Previous literature (6, 27) has reported increases in cerebral blood volume and oxygenation post-donation, further suggesting that cerebral and central compensatory responses are robust following mild hypovolemia. Our findings support and extend these observations by providing paired central and peripheral hemodynamic data in a real-world blood donation setting. Previous studies have shown that peripheral blood pressure decreases with blood donation, a result that was reflected in our cohort (20).

Moreover, in the acute post-donation window, a significant reduction in AIx and LVET was observed, indicating dynamic changes in ventricular–vascular coupling and wave reflection patterns. Another study demonstrated that reductions in preload—induced by head-up tilt—significantly decreased the AIx. Interestingly this was associated with increases in heartrate, DBP and systemic vascular resistance and a decrease in stroke volume (28). The absence of significant changes in heart rate and DBP despite a reduction in AIx in our study may be attributed to the more rapid hemodynamic challenge imposed by the head-up tilt test compared to blood donation. The slower onset of volume depletion during blood donation likely allows for more effective autonomic compensation, thereby attenuating immediate cardiovascular responses.

While cardiac output adapts in response to hypovolemia, arterial blood pressure is the primary variable sensed and regulated via arterial baroreceptors. Baroreflex activation promptly increases sympathetic outflow, which typically results in enhanced heart rate and peripheral vasoconstriction to maintain central pressure. In the cohort, no statistically significant increase in heart rate or direct evidence of peripheral vasoconstriction was observed, as assessed by model-based resistance indices and the notable decrease in AIx.

Instead, the data suggest a trend toward mild peripheral vasodilation post-donation. This could be attributable to the relatively modest magnitude of blood loss in standardized donation, a healthy, well-hydrated study population, or the short duration of measurement—factors that may preclude robust sympathetic activation. Additionally, the immediate increase in vagal tone or local vasodilatory factors cannot be excluded. We did not measure direct indices of peripheral perfusion (e.g., capillary refill time, skin temperature, or lactate levels), which limits our ability to fully assess microvascular responses—a limitation that should be addressed in future studies.

Regarding the analysis of correlations between baseline characteristics and hemodynamic changes, our intent was to explore whether age, sex, BMI, or hemoglobin modulate cardiovascular adaptation to acute blood loss. No statistically significant correlations emerged, and the effect sizes were minimal; these findings should be interpreted cautiously, especially given the modest sample size and the exploratory nature of these analyses. We acknowledge that inclusion of extensive statistical correlation analyses may risk overstating conclusions and have therefore restrained interpretation, confining these findings to descriptive context and providing visualizations in Supplementary Figures S1, S2.

The present sample size (*n* = 30) is comparable to that of several prior physiological studies of acute blood loss and is justified by the primary objective of detecting significant changes in arterial pressure. While the sample size limits the detection of subtle subgroup effects, it is adequate for the central question of the project. The sample composition also allows generalizability to a typical blood-donor population, which spans a broad adult age range but is not enriched for elderly or cardiovascularly vulnerable individuals.

There are important limitations to this work. First, the exclusive use of the VascAssist 2 device restricts interpretation, as reference data regarding its concordance with invasive assessments are limited. Previous validation studies (12) indicate that accuracy may vary as a function of absolute pressure, especially in hypotensive ranges, and caution is warranted when extrapolating to clinical populations. The absence of complementary hemodynamic modalities—such as echocardiographic measurements or direct tissue perfusion markers—limits characterization of the full spectrum of compensatory responses. Although invasive hemodynamic assessment would provide a gold standard, it is ethically inappropriate in this healthy donor context.

It must be acknowledged that the VascAssist 2 device, although validated in previous studies (12), employs a model-based approach which may underestimate absolute central pressures in hypotensive ranges. This device-related constraint, along with the exclusive reliance on oscillometric PWA, limits comparability with alternative modalities such as applanation tonometry or echocardiography.

## Conclusion

In conclusion, this study demonstrates that standard-volume whole blood donation in healthy adults produces a mild but statistically significant reduction in peripheral SBP, accompanied by a significant decrease in AIx and LVET, while central aortic hemodynamics remain largely preserved. These findings highlight the rapidity and robustness of physiological adaptations to mild, controlled hypovolemia and provide novel insights by utilizing continuous, operator-independent measurement techniques, which improve the precision and reliability of hemodynamic assessment. Importantly, the observed changes were consistent across participants, suggesting that the cardiovascular system responds uniformly to standard-volume blood loss in otherwise healthy individuals. The results have broader implications for understanding compensatory mechanisms during acute hypovolemic stress, potentially informing clinical practices in transfusion medicine, perioperative management, and cardiovascular risk assessment. Furthermore, our data underscore the value of non-invasive, high-resolution monitoring to detect subtle hemodynamic shifts that may not be apparent with conventional measurement methods. Future research should explore multi-modal hemodynamic assessments, including direct measures of peripheral perfusion, to better delineate the temporal dynamics and inter-individual variability of these responses. Expanding these investigations to populations with cardiovascular comorbidities or altered autonomic regulation may elucidate potential differences in compensatory capacity and inform personalized management strategies. Overall, the present study contributes to a more nuanced understanding of acute cardiovascular adaptation, bridging gaps in the literature and setting the stage for targeted follow-up studies.

## Data Availability

The original contributions presented in the study are included in the article/Supplementary Material, further inquiries can be directed to the corresponding author.

## Supplementary material

Supplementary Figure S1 Heart rate at rest for male vs. female participants. Bpm, beats per minute.

Supplementary Figure S2 Association of augmentation index adjusted to a heart rate of 75 beats per minute (AI×75) with body height.

Supplementary Table S1 AI×75, augmentation index adjusted to a heart rate of 75 beats per minute; AP, augmentation pressure; BP, blood pressure; bpm, beats per minute; DBP, diastolic blood pressure; LVET, left ventricular ejection time; PWV, pulse wave velocity; SBP, systolic blood pressure.
