# Supplementary figures and images for: Changes in central and peripheral hemodynamic parameters during blood donation
Source: Front Cardiovasc Med. 2025 Sep 23;12:1628366. doi: 10.3389/fcvm.2025.1628366 (PMC12500645; doi:10.3389/fcvm.2025.1628366)

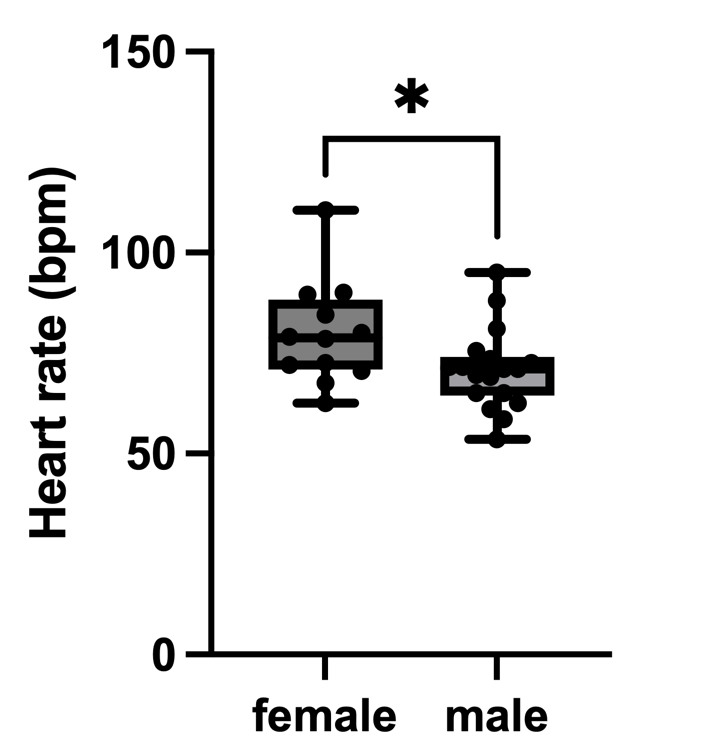

Supplement: Supplementary Figure S1 — Heart rate at rest for male vs. female participants. Bpm, beats per minute. [file Image1.tiff]

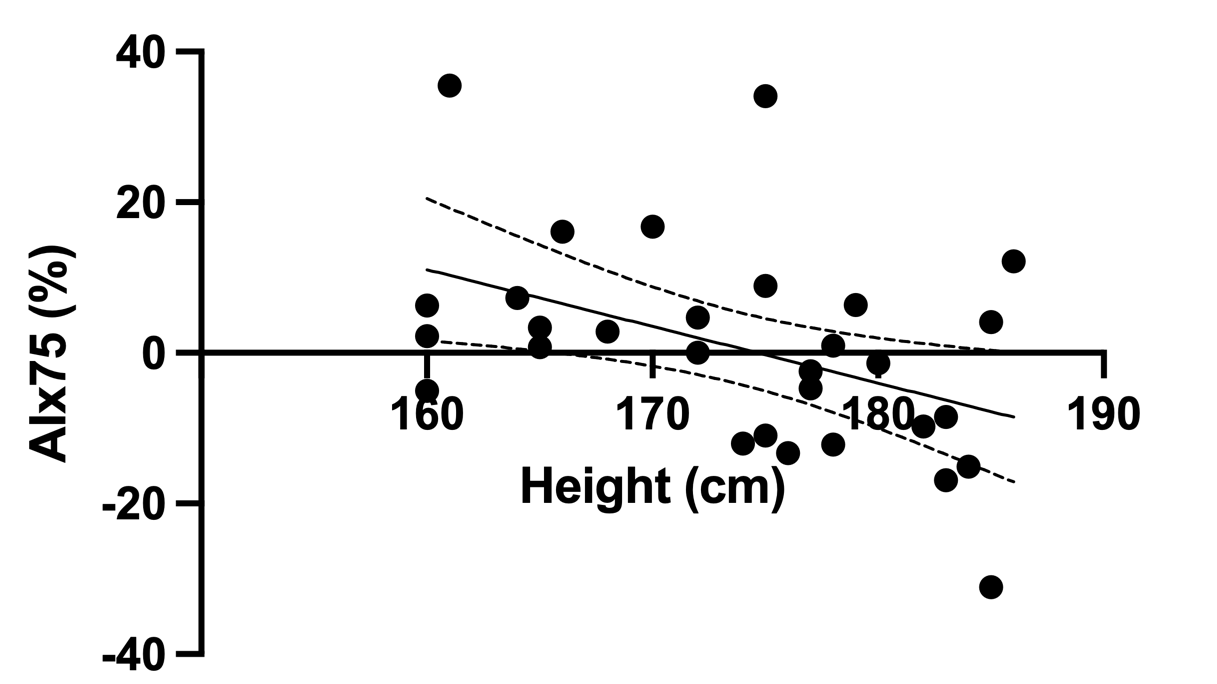

Supplement: Supplementary Figure S2 — Association of augmentation index adjusted to a heart rate of 75 beats per minute (AI×75) with body height. [file Image2.tiff]
