# Supplementary material for: Changes in central and peripheral hemodynamic parameters during blood donation
Source: Front Cardiovasc Med. 2025 Sep 23;12:1628366. doi: 10.3389/fcvm.2025.1628366 (PMC12500645; doi:10.3389/fcvm.2025.1628366)
Supplement: Supplementary Table S1 — AI×75, augmentation index adjusted to a heart rate of 75 beats per minute; AP, augmentation pressure; BP, blood pressure; bpm, beats per minute; DBP, diastolic blood pressure; LVET, left ventricular ejection time; PWV, pulse wave velocity; SBP, systolic blood pressure. [file Table1.pdf]

Supplementary Table 1.

	Pearson correlation coefficient r	95% CI	p-value
Sex			
Stiffness	-0.084	-0.431 to 0.285	0.658
Resistance	0.204	-0.169 to 0.526	0.280
Aortic SBP	0.197	-0.176 to 0.520	0.298
Aortic DBP	0.273	-0.097 to 0.576	0.144
AIx75	0.188	-0.185 to 0.514	0.320
Heart rate	0.097	-0.273 to 0.442	0.612
Brachial SBP	0.147	-0.226 to 0.481	0.440
Brachial DBP	0.222	-0.151 to 0.539	0.240
LVET	0.174	-0.199 to 0.503	0.359
Central PWV	0.174	-0.199 to 0.503	0.359
Age			
Stiffness	0.029	-0.335 to 0.386	0.878
Resistance	-0.055	-0.407 to 0.312	0.774
Aortic SBP	0.258	-0.113 to 0.565	0.169
Aortic DBP	0.012	-0.350 to 0.371	0.950
AIx75	-0.175	-0.504 to 0.198	0.355
Heart rate	0.362	0.002 to 0.639	0.050
Brachial SBP	0.351	-0.011 to 0.631	0.057
Brachial DBP	0.070	-0.298 to 0.420	0.714
LVET	0.080	-0.288 to 0.428	0.674
Central PWV	0.089	-0.280 to 0.435	0.640
BMI			
Stiffness	0.220	-0.152 to 0.538	0.242
Resistance	0.178	-0.195 to 0.505	0.348
Aortic SBP	-0.250	-0.560 to 0.121	0.182
Aortic DBP	-0.240	-0.553 to 0.131	0.201
AIx75	-0.006	-0.366 to 0.355	0.974
Heart rate	0.088	-0.281 to 0.435	0.644
Brachial SBP	-0.118	-0.459 to 0.253	0.535
Brachial DBP	-0.158	-0.491 to 0.214	0.404
LVET	-0.086	-0.433 to 0.283	0.653
Central PWV	0.159	-0.214 to 0.491	0.403
Hemoglobin			
Stiffness	0.252	-0.119 to 0.562	0.179
Resistance	-0.024	-0.381 to 0.340	0.901
Aortic SBP	-0.176	-0.504 to 0.197	0.352
Aortic DBP	-0.097	-0.442 to 0.273	0.610
AIx75	0.008	-0.353 to 0.368	0.965
Heart rate	-0.003	-0.363 to 0.358	0.987
Brachial SBP	-0.352	-0.632 to 0.009	0.056
Brachial DBP	-0.079	-0.427 to 0.290	0.680
LVET	0.102	-0.268 to 0.446	0.590
Central PWV	0.102	-0.268 to 0.446	0.590

AIx75, augmentation index adjusted to a heart rate of 75 beats per minute; AP, augmentation pressure; BP, blood pressure; bpm, beats per minute; DBP, diastolic blood pressure; LVET, left ventricular ejection time; PWV, pulse wave velocity; SBP, systolic blood pressure.
